# Coach-Supported Internet-Based Cognitive Therapy for Social Anxiety Disorder in Hong Kong: Development, Training, and Pilot Clinical Outcomes

**DOI:** 10.2196/69818

**Published:** 2025-08-26

**Authors:** Graham R Thew, Candice LYM Powell, Lok Sum Chan, Ezmond Siu Long Cheung, Jeff Lau Tsz Chung, Chun Lap Li, Melody Miriam So, Yik Kwan Hsu, Man Ying Ng, Sze Hang Siu, Katja Beardsell, Amy PL Kwok, Mandy H Lissillour Chan, David M Clark, Patrick WL Leung

**Affiliations:** 1 Oxford Health National Health Service Foundation Trust, Oxford, United Kingdom; 2 Department of Experimental Psychology, University of Oxford, The Old Rectory, Paradise Square, Oxford, OX1 1TW, United Kingdom, 44 1865 281607; 3 Mind Hong Kong, Hong Kong, China; 4 Department of Psychology, Chinese University of Hong Kong, Hong Kong, China; 5 Department of Clinical Psychology, Hong Kong East Cluster, Hospital Authority, Hong Kong, China

**Keywords:** social anxiety disorder, cognitive behavioral therapy, training, supervision, internet interventions

## Abstract

Internet-based Cognitive Therapy for Social Anxiety Disorder (iCT-SAD), a treatment that is normally supported by therapists, has been modified for delivery by trained coaches without mental health practitioner qualifications and has shown promising efficacy for patients in Hong Kong.

## Introduction

Internet-based Cognitive Therapy for Social Anxiety Disorder (iCT-SAD), a therapist-guided treatment based on the social anxiety disorder (SAD) model of Clark and Wells [1], was designed to replicate the face-to-face treatment protocol and has shown good efficacy in the United Kingdom [2,3] and Hong Kong [4,5]. Clinical psychologists provided guidance in these studies. Other internet interventions have shown positive outcomes using guidance from trained coaches [6]. Coach support may be less resource-intensive and more scalable, but it is unknown whether coaches can effectively deliver iCT-SAD, which typically has close therapist support.

This letter describes the development of a modified version of iCT-SAD, optimised for delivery by coaches, along with coach training and supervision, and pilot patient outcomes.

## Methods

### Design

This single-cohort pilot study measured social anxiety symptoms weekly using the self-reported Liebowitz Social Anxiety Scale (LSAS) [7].

### Intervention

iCT-SAD includes core modules on key SAD treatment principles and a range of optional modules for tailoring treatment to patients’ specific concerns [2,3]. Support is provided via telephone, messaging, and SMS texts. Four calls are scheduled over the first 2 weeks, then weekly thereafter until week 14. Three monthly booster calls are then scheduled to monitor and consolidate progress. Behavioral experiments, constituting practical exercises to test fearful beliefs in anxiety-provoking situations [8], are used throughout treatment. The English-language program was translated into Chinese following a similar process for a Japanese translation [9].

We significantly modified 3 of 27 iCT-SAD modules for coach support by replacing or removing elements requiring expert therapist support. Typically, therapists support patients in completing exercises speaking to a stranger via webcam, demonstrating experientially the disadvantages of self-focused attention and safety behaviors [10], with subsequent video feedback [11]. These two modules were replaced with a demonstration video and guidance on implementing this experiment independently. The module addressing past socially traumatic memories was edited to focus solely on stimulus discrimination, removing imagery rescripting, which typically requires closer therapist guidance. We added new videos (reducing safety behaviors, generating experiment ideas, behavioral experiment tips) and made minor text changes to reflect the coach role (removing mention of the therapist administering surveys, which was not within the coach role). The frequency and length of coach phone calls were identical to therapist calls in previous studies.

### Coach Recruitment, Training, and Supervision

Five psychology graduates with no professional qualifications in mental health care (2 female, 3 male; mean age 25.0, SD 1.22 years) were recruited as coaches. Training consisted of a 1-day in-person workshop, followed by two 2-hour online training sessions given by GRT. These were adapted from previous iCT-SAD training [4] and covered key principles of SAD and cognitive therapy, the treatment procedure, the website, and patient communication methods. The training summarized each module, focusing on how coaches support patients within their role. It emphasized taking queries to their supervisor and fidelity to the treatment content and training.

Coaches then received weekly group supervision with CLYMP (a clinical psychologist) as they saw initial cases. Each saw 2 to 3 cases between September 2023 and July 2024.

### Participants

Twelve participants received treatment (10 female, 2 male; mean age 26.3, SD 4.1 years). All were referred from clinical services and experienced SAD as their main concern, confirmed at assessment by a clinical psychologist. All had sufficient literacy, time, and technology to undertake treatment.

### Ethical Considerations

The Joint Chinese University of Hong Kong-New Territories East Cluster Clinical Research Ethics Committee approved this study (2022.086-T). All participants provided informed consent, which included anonymized reporting of outcomes.

## Results

**Figure 1.**
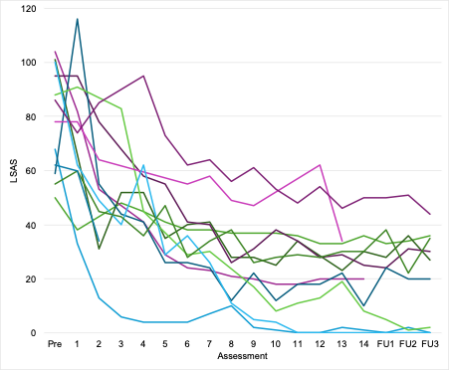
Liebowitz Social Anxiety Scale (LSAS): weekly (1-14) and monthly booster follow-up [FU] 1-3) scores for each participant in the pilot treatment cohort.

Ten participants completed the 14-week treatment phase and 9 completed the monthly booster phase. One participant withdrew in week 2, reporting treatment did not suit them, 1 withdrew in week 13 due to work commitments, and 1 was uncontactable during the booster phase. Participants spent a mean of 22.2 (SD 14.4) hours on the site. They were granted access to a mean of 19.6 (SD 4.7) modules, and completed a mean of 18.2 (SD 5.8;93%), plus a mean of 12.6 (SD 12.3) behavioral experiments. Figure 1 shows weekly LSAS scores [7], indicating substantial reductions in SAD symptoms. The mean scores were 78.8 (SD 19.5) at baseline and 23.2 (SD 15.5) at week 14. A pre-post paired 2-tailed *t* test was significant *(t*_11_=7.18; *P*<.001). The effect size estimate (Cohen *d*) was 2.86, which is considered large.

## Discussion

The findings provide initial evidence that coach-supported iCT-SAD can achieve positive outcomes broadly consistent with Thew et al [5]. Further text modifications and updates to coach training (eg, to clarify task instructions and emphasize video feedback) have been made where indicated. Findings are limited by the lack of a control group and the small sample, but progression to a larger randomized controlled trial is indicated to evaluate it against waitlist and active controls.

## Abbreviations

iCT-SAD: Internet-based Cognitive Therapy for Social Anxiety Disorder
LSAS: Liebowitz Social Anxiety Scale
SAD: social anxiety disorder
